# 6-Bromo-3-methyl-1*H*-imidazo[4,5-*b*]pyridin-2(3*H*)-one

**DOI:** 10.1107/S1600536810012791

**Published:** 2010-04-14

**Authors:** Hend Bel Ghacham, Youssef Kandri Rodi, Frédéric Capet, El Mokhtar Essassi, Seik Weng Ng

**Affiliations:** a Laboratoire de Chimie Organique Appliquée, Faculté des Sciences et Techniques, Université Sidi Mohamed Ben Abdallah, Fés, Morocco; b Unité de Catalyse et de Chimie du Solide (UCCS), UMR 8181, Ecole Nationale Supérieure de Chimie de Lille, Lille, France; c Laboratoire de Chimie Organique Hétérocyclique, Pôle de Compétences Pharmacochimie, Université Mohammed V-Agdal, BP 1014 Avenue Ibn Batout, Rabat, Morocco; d Department of Chemistry, University of Malaya, 50603 Kuala Lumpur, Malaysia

## Abstract

The title compound, C_7_H_6_BrN_3_O, was obtained from the reaction of 6-bromo-1*H*-imidazo[4,5-*b*]pyridin-2(3*H*)-one with methyl iodide. All non-H atoms lie in a common plane [r.m.s deviation = 0.017 (1) Å]. The amino group is a hydrogen-bond donor to the carbonyl group of an inversion-related mol­ecule, the pair of hydrogen bonds giving rise to a hydrogen-bonded dimer.

## Related literature

For the synthesis of the title compound, see: Grivas & Lindström (1995 ▶); Smolyar *et al.* (2007 ▶).
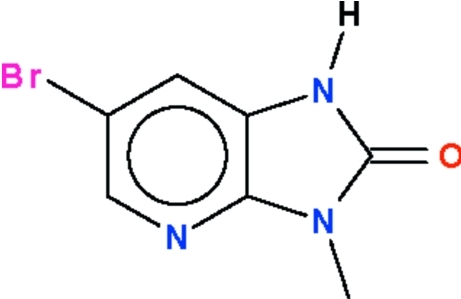


## Experimental

### 

#### Crystal data

C_7_H_6_BrN_3_O
*M* *_r_* = 228.06
Triclinic, 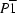

*a* = 4.4151 (1) Å
*b* = 9.6004 (2) Å
*c* = 10.5330 (3) Å
α = 116.248 (1)°
β = 93.074 (2)°
γ = 91.687 (1)°
*V* = 399.14 (2) Å^3^
*Z* = 2
Mo *K*α radiation
μ = 5.10 mm^−1^
*T* = 293 K
0.36 × 0.17 × 0.10 mm

#### Data collection

Bruker X8 APEXII diffractometer
Absorption correction: multi-scan (*SADABS*; Sheldrick, 1996 ▶) *T*_min_ = 0.478, *T*_max_ = 0.630
4790 measured reflections
1401 independent reflections
1199 reflections with *I* > 2σ(*I*)
*R*_int_ = 0.027
Standard reflections: 0

#### Refinement

*R*[*F* ^2^ > 2σ(*F* ^2^)] = 0.030
*wR*(*F* ^2^) = 0.077
*S* = 1.05
1401 reflections
114 parameters
1 restraint
H atoms treated by a mixture of independent and constrained refinement
Δρ_max_ = 0.52 e Å^−3^
Δρ_min_ = −0.32 e Å^−3^

### 

Data collection: *APEX2* (Bruker, 2008 ▶); cell refinement: *SAINT* (Bruker, 2008 ▶); data reduction: *SAINT*; program(s) used to solve structure: *SHELXS97* (Sheldrick, 2008 ▶); program(s) used to refine structure: *SHELXL97* (Sheldrick, 2008 ▶); molecular graphics: *X-SEED* (Barbour, 2001 ▶); software used to prepare material for publication: *publCIF* (Westrip, 2010 ▶).

## Supplementary Material

Crystal structure: contains datablocks global, I. DOI: 10.1107/S1600536810012791/bt5243sup1.cif

Structure factors: contains datablocks I. DOI: 10.1107/S1600536810012791/bt5243Isup2.hkl

Additional supplementary materials:  crystallographic information; 3D view; checkCIF report

## Figures and Tables

**Table 1:** Hydrogen-bond geometry (Å, °)

*D*—H⋯*A*	*D*—H	H⋯*A*	*D*⋯*A*	*D*—H⋯*A*
N3—H3⋯O1^i^	0.86 (1)	1.95 (1)	2.804 (3)	176 (4)

Symmetry code: (i) 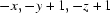.

**Fig. 1.**
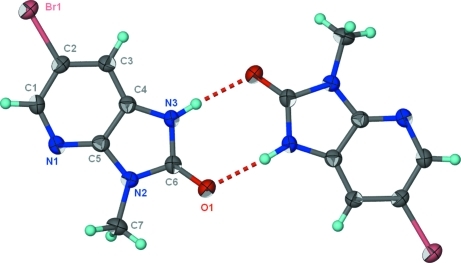
Anisotropic displacement ellipsoid plot (Barbour, 2001) of the title compound forming a centrosymmetric dimer at the 50% probability level; H atoms are drawn as spheres of arbitrary radii.

## supplementary crystallographic information

### Comment

6-Bromo-1*H*-imidazo[4,5-*b*]pyridine-2(3*H*)-one reacts with organic compounds to form pharmaceutical active compounds. It is easily methylated; in this study, it is methylated by methyl iodide under catalytic conditions. The mono *N*-methylated compound (Scheme I) is planar [r.m.s 0.017 (1) Å]. The amino group is hydrogen-bond donor to the carbonyl group of an inversion-related molecule to generate a hydrogen-bonded dimer (Fig. 1).

### Experimental

6-Bromo-1*H*-imidazo[4,5-*b*]pyridine-2(3*H*)-thione (1 mmol), potassium carbonate (4 mmol), tetra-*n*-butylammonium bromide (0.1 mmol) and methyl iodide (2.5 mmol) in DMF (15 ml) were stirred for 48 hours. After completion of reaction (as monitored by TLC), the salt was filtered and the solvent removed under reduced pressure. The resulting residue was purified by column chromatography on silica gel using ethyl acetate/hexane (1/2) as eluent. Yellow crystals was isolated when the solvent was allowed to evaporate.

### Refinement

Carbon-bound H atoms were placed in calculated positions (C—H = 0.93–0.96 Å) and were included in the refinement in the riding model approximation, with *U*(H) set to 1.2*U*(C). The amino H atom was located in a difference Fourier map, and was refined isotropically with a distance restraint of N—H = 0.86 (1) Å.

### Crystal data

**Table:**

C_7_H_6_BrN_3_O	*Z* = 2
*M**_r_* = 228.06	*F*(000) = 224
Triclinic, *P*1	*D*_x_ = 1.898 Mg m^−^^3^
Hall symbol: -P 1	Mo *K*α radiation, λ = 0.71073 Å
*a* = 4.4151 (1) Å	Cell parameters from 2054 reflections
*b* = 9.6004 (2) Å	θ = 2.4–24.1°
*c* = 10.5330 (3) Å	µ = 5.10 mm^−^^1^
α = 116.248 (1)°	*T* = 293 K
β = 93.074 (2)°	Prism, yellow
γ = 91.687 (1)°	0.36 × 0.17 × 0.10 mm
*V* = 399.14 (2) Å^3^	

### Data collection

**Table:**

Bruker X8 APEXII diffractometer	1401 independent reflections
Radiation source: fine-focus sealed tube	1199 reflections with *I* > 2σ(*I*)
graphite	*R*_int_ = 0.027
φ and ω scans	θ_max_ = 24.9°, θ_min_ = 2.2°
Absorption correction: multi-scan (*SADABS*; Sheldrick, 1996)	*h* = −5→4
*T*_min_ = 0.478, *T*_max_ = 0.630	*k* = −11→11
4790 measured reflections	*l* = −12→12

### Refinement

**Table:**

Refinement on *F*^2^	Primary atom site location: structure-invariant direct methods
Least-squares matrix: full	Secondary atom site location: difference Fourier map
*R*[*F*^2^ > 2σ(*F*^2^)] = 0.030	Hydrogen site location: inferred from neighbouring sites
*wR*(*F*^2^) = 0.077	H atoms treated by a mixture of independent and constrained refinement
*S* = 1.05	*w* = 1/[σ^2^(*F*_o_^2^) + (0.0493*P*)^2^ + 0.0118*P*] where *P* = (*F*_o_^2^ + 2*F*_c_^2^)/3
1401 reflections	(Δ/σ)_max_ = 0.001
114 parameters	Δρ_max_ = 0.52 e Å^−^^3^
1 restraint	Δρ_min_ = −0.32 e Å^−^^3^

### Fractional atomic coordinates and isotropic or equivalent isotropic displacement parameters (Å^2^)

**Table:**

	*x*	*y*	*z*	*U*_iso_*/*U*_eq_	
Br1	0.91934 (8)	0.96903 (4)	0.29671 (4)	0.04832 (17)	
O1	0.0726 (5)	0.3085 (3)	0.3562 (3)	0.0478 (6)	
N1	0.7410 (6)	0.4941 (3)	0.1298 (3)	0.0393 (6)	
N2	0.4091 (6)	0.3574 (3)	0.2169 (3)	0.0368 (6)	
N3	0.2638 (6)	0.5575 (3)	0.4045 (3)	0.0373 (6)	
H3	0.165 (7)	0.603 (4)	0.478 (2)	0.052 (11)*	
C1	0.8436 (8)	0.6389 (4)	0.1562 (3)	0.0395 (8)	
H1	0.9737	0.6506	0.0946	0.047*	
C2	0.7621 (7)	0.7715 (4)	0.2724 (3)	0.0362 (7)	
C3	0.5682 (7)	0.7635 (3)	0.3680 (3)	0.0357 (7)	
H3A	0.5157	0.8517	0.4462	0.043*	
C4	0.4584 (7)	0.6171 (4)	0.3399 (3)	0.0332 (7)	
C5	0.5521 (7)	0.4890 (4)	0.2200 (3)	0.0326 (7)	
C6	0.2306 (7)	0.3988 (4)	0.3293 (3)	0.0359 (7)	
C7	0.4409 (9)	0.1982 (4)	0.1123 (4)	0.0532 (10)	
H7A	0.6141	0.1577	0.1406	0.080*	
H7B	0.4681	0.1960	0.0216	0.080*	
H7C	0.2613	0.1357	0.1055	0.080*	

### Atomic displacement parameters (Å^2^)

**Table:**

	*U*^11^	*U*^22^	*U*^33^	*U*^12^	*U*^13^	*U*^23^
Br1	0.0551 (3)	0.0388 (2)	0.0537 (3)	−0.00649 (16)	0.00846 (17)	0.02302 (18)
O1	0.0560 (15)	0.0356 (12)	0.0502 (14)	−0.0057 (11)	0.0173 (12)	0.0168 (11)
N1	0.0399 (15)	0.0385 (15)	0.0382 (15)	0.0040 (12)	0.0119 (12)	0.0146 (13)
N2	0.0420 (15)	0.0298 (13)	0.0356 (14)	0.0018 (11)	0.0087 (12)	0.0112 (11)
N3	0.0406 (16)	0.0336 (14)	0.0361 (15)	0.0011 (12)	0.0140 (13)	0.0131 (12)
C1	0.0404 (18)	0.0410 (18)	0.0380 (18)	0.0024 (14)	0.0119 (15)	0.0175 (16)
C2	0.0372 (17)	0.0328 (16)	0.0416 (19)	−0.0016 (13)	0.0022 (15)	0.0194 (15)
C3	0.0367 (17)	0.0305 (16)	0.0381 (17)	0.0040 (13)	0.0074 (14)	0.0131 (14)
C4	0.0294 (16)	0.0336 (16)	0.0353 (17)	0.0032 (13)	0.0029 (13)	0.0139 (14)
C5	0.0305 (16)	0.0308 (15)	0.0344 (16)	0.0030 (13)	0.0015 (13)	0.0126 (13)
C6	0.0349 (17)	0.0367 (17)	0.0357 (17)	0.0011 (14)	0.0064 (14)	0.0155 (14)
C7	0.067 (3)	0.0341 (18)	0.049 (2)	0.0033 (17)	0.0166 (19)	0.0079 (16)

### Geometric parameters (Å, °)

**Table:**

Br1—C2	1.902 (3)	C1—C2	1.394 (5)
O1—C6	1.233 (4)	C1—H1	0.9300
N1—C5	1.313 (4)	C2—C3	1.382 (4)
N1—C1	1.349 (4)	C3—C4	1.369 (4)
N2—C6	1.373 (4)	C3—H3A	0.9300
N2—C5	1.382 (4)	C4—C5	1.411 (4)
N2—C7	1.452 (4)	C7—H7A	0.9600
N3—C6	1.370 (4)	C7—H7B	0.9600
N3—C4	1.381 (4)	C7—H7C	0.9600
N3—H3	0.856 (10)		
			
C5—N1—C1	114.5 (3)	C3—C4—N3	134.7 (3)
C6—N2—C5	109.8 (2)	C3—C4—C5	118.7 (3)
C6—N2—C7	124.2 (3)	N3—C4—C5	106.6 (3)
C5—N2—C7	126.1 (3)	N1—C5—N2	126.7 (3)
C6—N3—C4	110.0 (3)	N1—C5—C4	126.5 (3)
C6—N3—H3	119 (3)	N2—C5—C4	106.7 (3)
C4—N3—H3	131 (3)	O1—C6—N3	127.3 (3)
N1—C1—C2	122.5 (3)	O1—C6—N2	125.8 (3)
N1—C1—H1	118.7	N3—C6—N2	106.9 (3)
C2—C1—H1	118.7	N2—C7—H7A	109.5
C3—C2—C1	122.1 (3)	N2—C7—H7B	109.5
C3—C2—Br1	119.5 (2)	H7A—C7—H7B	109.5
C1—C2—Br1	118.4 (2)	N2—C7—H7C	109.5
C4—C3—C2	115.6 (3)	H7A—C7—H7C	109.5
C4—C3—H3A	122.2	H7B—C7—H7C	109.5
C2—C3—H3A	122.2		
			
C5—N1—C1—C2	1.8 (5)	C6—N2—C5—C4	0.2 (3)
N1—C1—C2—C3	−0.5 (5)	C7—N2—C5—C4	−179.4 (3)
N1—C1—C2—Br1	−179.4 (2)	C3—C4—C5—N1	0.6 (5)
C1—C2—C3—C4	−0.9 (5)	N3—C4—C5—N1	−179.2 (3)
Br1—C2—C3—C4	178.0 (2)	C3—C4—C5—N2	−179.9 (3)
C2—C3—C4—N3	−179.4 (3)	N3—C4—C5—N2	0.3 (3)
C2—C3—C4—C5	0.8 (4)	C4—N3—C6—O1	−179.5 (3)
C6—N3—C4—C3	179.6 (3)	C4—N3—C6—N2	0.7 (4)
C6—N3—C4—C5	−0.6 (3)	C5—N2—C6—O1	179.7 (3)
C1—N1—C5—N2	178.8 (3)	C7—N2—C6—O1	−0.8 (5)
C1—N1—C5—C4	−1.9 (5)	C5—N2—C6—N3	−0.5 (3)
C6—N2—C5—N1	179.6 (3)	C7—N2—C6—N3	179.0 (3)
C7—N2—C5—N1	0.1 (5)		

### Hydrogen-bond geometry (Å, °)

**Table:**

*D*—H···*A*	*D*—H	H···*A*	*D*···*A*	*D*—H···*A*
N3—H3···O1^i^	0.86 (1)	1.95 (1)	2.804 (3)	176 (4)

Symmetry codes: (i) −*x*, −*y*+1, −*z*+1.
